# 4-{2-[5-(3,5-Difluoro­phen­yl)-2-methyl­thio­phen-3-yl]-3,3,4,4,5,5-hexa­fluoro­cyclo­pent-1-en-1-yl}-1,5-dimethyl­pyrrole-2-carbonitrile

**DOI:** 10.1107/S1600536811009780

**Published:** 2011-03-23

**Authors:** Gang Liu, Xiao-mei Wang, Cong-bin Fan

**Affiliations:** a College of Chemistry, Chemical Engineering and Materials Science, Soochow University, Suzhou 215123, People’s Republic of China

## Abstract

In the title compound, C_23_H_14_F_8_N_2_S, the dihedral angles between the pyrrole and thio­phene groups and the almost planar C—C=C—C unit of the cyclo­pentene ring (r.m.s. deviation = 0.4193 Å) are 43.6 (5) and 50.1 (2)°, respectively. The distance of 3.612 (3) Å between the potentially reactive C atoms of the two heteroaryl substituents is short enough to enable a photocyclization reaction.

## Related literature

The title compound belongs to a new family of organic photochromic diaryl­ethene compounds with an unsymmetrically substituted hexa­fluoro­cyclo­pentene unit. For background to these compounds, see: Pu *et al.* (2007 ▶); Liu *et al.* (2011 ▶). For details of the synthesis, see: Fan *et al.* (2011 ▶).
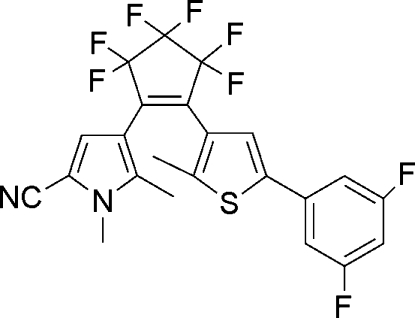


## Experimental

### 

#### Crystal data

C_23_H_14_F_8_N_2_S
*M* *_r_* = 502.42
Monoclinic, 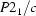

*a* = 11.873 (2) Å
*b* = 12.063 (2) Å
*c* = 16.208 (3) Å
β = 109.225 (3)°
*V* = 2191.9 (7) Å^3^
*Z* = 4
Mo *K*α radiation
μ = 0.23 mm^−1^
*T* = 294 K
0.24 × 0.20 × 0.12 mm

#### Data collection

Bruker SMART CCD area-detector diffractometer
Absorption correction: multi-scan (*SADABS*; Sheldrick, 1996 ▶) *T*_min_ = 0.947, *T*_max_ = 0.973
10859 measured reflections
3870 independent reflections
2075 reflections with *I* > 2σ(*I*)
*R*_int_ = 0.052

#### Refinement

*R*[*F* ^2^ > 2σ(*F* ^2^)] = 0.045
*wR*(*F* ^2^) = 0.108
*S* = 1.00
3870 reflections
310 parameters
H-atom parameters constrained
Δρ_max_ = 0.22 e Å^−3^
Δρ_min_ = −0.23 e Å^−3^

### 

Data collection: *SMART* (Bruker, 1997 ▶); cell refinement: *SAINT* (Bruker, 1997 ▶); data reduction: *SAINT*; program(s) used to solve structure: *SHELXS97* (Sheldrick, 2008 ▶); program(s) used to refine structure: *SHELXL97* (Sheldrick, 2008 ▶); molecular graphics: *SHELXTL* (Sheldrick, 2008 ▶); software used to prepare material for publication: *SHELXTL*.

## Supplementary Material

Crystal structure: contains datablocks global, I. DOI: 10.1107/S1600536811009780/gk2352sup1.cif

Structure factors: contains datablocks I. DOI: 10.1107/S1600536811009780/gk2352Isup2.hkl

Additional supplementary materials:  crystallographic information; 3D view; checkCIF report

## Figures and Tables

**Fig. 1.**
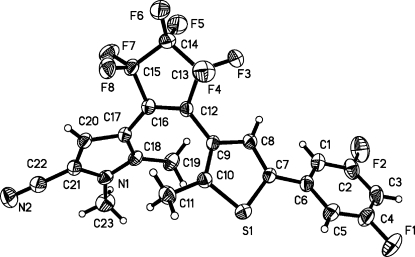
Molecular structure of the title compound with displacement ellipsoids drawn at the 30% probability level. H atoms are represented as small spheres of arbitrary radii.

## supplementary crystallographic information

### Comment

The title compound when dissolved in hexane shows photochromism. Upon irradiation with 365 nm light, the colorless  hexane solution turns blue rapidly. The blue compound displays an absorption maximum at 592 nm. Upon irradiation with visible light with wavelength longer than 510 nm, the blue hexane solution reverts to its initial colorless state; a colorless hexane solution of the title compound has two absorption maximum at 253 nm and 294 nm. In a polymethylmethacrylate amorphous film, the title diarylethene also exhibits photochromism similar to that in hexane.

### Experimental

To a tetrahydrofuran solution of 1-bromo-3,5-difluorobenzene (1.93 g, 10.0 mmol) was added 3-bromo-2-methyl-5-thienylboronic acid (2.50 g, 11.3 mmol) (Fan *et al.*, 2011) in the presence of Pd(PPh_3_)_4_ (0.3 g) and Na_2_CO_3_ (6.4 g, 60 mmol) in 20 ml H_2_O. After refluxing for 15 h, the product, 3-Bromo-2-methyl-5-(3,5-difluorophenyl)thiophene (1.94 g, 6.73 mmol), was collected and dried (yield 67.3%). This compound (0.67 g, 2.3 mmol) was reacted with 1-(2-cyano-1,5-dimethyl-4-pyrrol-1-yl)-3,3,4,4,5,5- hexafluorocyclopent-1-ene (0.66 g, 2.30 mmol)(Liu *et al.*, 2011) and with *n*-butyl lithium 2.5 *M* in hexane (0.92 ml, 2.30 mmol) at 195 K under a nitrogen atmosphere. After an hour, the reaction was quenched by addition of water. The solid product was purified by column chromatography on silica with petroleum ether as the eluent to give the title compound (0.55 g, 1.10 mmol) in 47.8% yield. Analysis calc. for C_23_H_14_F_8_N_2_S: C 54.98, H, 2.81%; fFound C 55.02, H 2.95%.

### Refinement

All H atoms were placed in calculated positionswith C—H equal 0.93 Å for aromatic and 0.96 Å for CH_3 _groups. They were included in the refinement in the riding model approximation with isotropic displacement parameters set equal to 1.2*U*_eq_(C) and 1.5*U*_eq_(C) of the carrier atom for the aromatic and methyl H atoms, respectively.

### Crystal data

**Table:**

C_23_H_14_F_8_N_2_S	*F*(000) = 1016
*M**_r_* = 502.42	*D*_x_ = 1.523 Mg m^−^^3^
Monoclinic, *P*2_1_/*c*	Mo *K*α radiation, λ = 0.71073 Å
Hall symbol: -P 2ybc	Cell parameters from 1969 reflections
*a* = 11.873 (2) Å	θ = 2.2–21.0°
*b* = 12.063 (2) Å	µ = 0.23 mm^−^^1^
*c* = 16.208 (3) Å	*T* = 294 K
β = 109.225 (3)°	Block, colourless
*V* = 2191.9 (7) Å^3^	0.24 × 0.20 × 0.12 mm
*Z* = 4	

### Data collection

**Table:**

Bruker SMART CCD area-detector diffractometer	3870 independent reflections
Radiation source: fine-focus sealed tube	2075 reflections with *I* > 2σ(*I*)
graphite	*R*_int_ = 0.052
φ and ω scans	θ_max_ = 25.0°, θ_min_ = 1.8°
Absorption correction: multi-scan (*SADABS*; Sheldrick, 1996)	*h* = −13→14
*T*_min_ = 0.947, *T*_max_ = 0.973	*k* = −14→7
10859 measured reflections	*l* = −19→17

### Refinement

**Table:**

Refinement on *F*^2^	Primary atom site location: structure-invariant direct methods
Least-squares matrix: full	Secondary atom site location: difference Fourier map
*R*[*F*^2^ > 2σ(*F*^2^)] = 0.045	Hydrogen site location: inferred from neighbouring sites
*wR*(*F*^2^) = 0.108	H-atom parameters constrained
*S* = 1.00	*w* = 1/[σ^2^(*F*_o_^2^) + (0.0294*P*)^2^ + 1.1725*P*] where *P* = (*F*_o_^2^ + 2*F*_c_^2^)/3
3870 reflections	(Δ/σ)_max_ < 0.001
310 parameters	Δρ_max_ = 0.22 e Å^−^^3^
0 restraints	Δρ_min_ = −0.23 e Å^−^^3^

### Special details

**Table:**

Geometry. All esds (except the esd in the dihedral angle between two l.s. planes) are estimated using the full covariance matrix. The cell esds are taken into account individually in the estimation of esds in distances, angles and torsion angles; correlations between esds in cell parameters are only used when they are defined by crystal symmetry. An approximate (isotropic) treatment of cell esds is used for estimating esds involving l.s. planes.
Refinement. Refinement of F^2^ against ALL reflections. The weighted R-factor wR and goodness of fit S are based on F^2^, conventional R-factors R are based on F, with F set to zero for negative F^2^. The threshold expression of F^2^ > 2sigma(F^2^) is used only for calculating R-factors(gt) etc. and is not relevant to the choice of reflections for refinement. R-factors based on F^2^ are statistically about twice as large as those based on F, and R- factors based on ALL data will be even larger.

### Fractional atomic coordinates and isotropic or equivalent isotropic displacement parameters (Å^2^)

**Table:**

	*x*	*y*	*z*	*U*_iso_*/*U*_eq_	
S1	0.20592 (8)	0.64459 (8)	0.08798 (6)	0.0544 (3)	
F1	−0.1023 (3)	0.5715 (2)	−0.22785 (15)	0.1251 (10)	
F2	−0.3477 (2)	0.7314 (2)	−0.08674 (16)	0.1138 (9)	
F3	0.12870 (18)	1.04692 (16)	0.16690 (13)	0.0782 (7)	
F4	0.31802 (19)	1.04374 (18)	0.19007 (13)	0.0794 (7)	
F5	0.1543 (2)	1.10471 (19)	0.32403 (14)	0.0904 (7)	
F6	0.3169 (2)	1.17628 (17)	0.31582 (13)	0.0908 (8)	
F7	0.3056 (2)	1.01110 (17)	0.45684 (13)	0.0798 (7)	
F8	0.44533 (17)	1.01423 (16)	0.39904 (12)	0.0738 (6)	
N1	0.2949 (2)	0.6232 (2)	0.45199 (17)	0.0506 (7)	
N2	0.5482 (3)	0.5675 (3)	0.6326 (2)	0.0928 (12)	
C1	−0.1419 (3)	0.7236 (3)	−0.0150 (2)	0.0584 (10)	
H1	−0.1522	0.7596	0.0328	0.070*	
C2	−0.2385 (3)	0.7008 (4)	−0.0874 (3)	0.0714 (12)	
C3	−0.2293 (4)	0.6494 (3)	−0.1595 (3)	0.0772 (13)	
H3	−0.2957	0.6352	−0.2082	0.093*	
C4	−0.1168 (4)	0.6197 (3)	−0.1564 (3)	0.0771 (12)	
C5	−0.0167 (3)	0.6391 (3)	−0.0855 (2)	0.0630 (10)	
H5	0.0580	0.6165	−0.0860	0.076*	
C6	−0.0287 (3)	0.6927 (3)	−0.0134 (2)	0.0485 (9)	
C7	0.0764 (3)	0.7213 (3)	0.06179 (19)	0.0442 (8)	
C8	0.0902 (3)	0.8070 (3)	0.11854 (19)	0.0477 (9)	
H8	0.0303	0.8583	0.1150	0.057*	
C9	0.2049 (3)	0.8111 (3)	0.18408 (18)	0.0433 (8)	
C10	0.2779 (3)	0.7270 (3)	0.17555 (19)	0.0459 (8)	
C11	0.4044 (3)	0.7020 (3)	0.2296 (2)	0.0637 (10)	
H11A	0.4424	0.7685	0.2578	0.096*	
H11B	0.4464	0.6739	0.1925	0.096*	
H11C	0.4053	0.6475	0.2729	0.096*	
C12	0.2404 (2)	0.8972 (3)	0.25172 (19)	0.0425 (8)	
C13	0.2332 (3)	1.0168 (3)	0.2267 (2)	0.0513 (9)	
C14	0.2579 (3)	1.0803 (3)	0.3117 (2)	0.0552 (9)	
C15	0.3261 (3)	0.9968 (3)	0.3807 (2)	0.0515 (9)	
C16	0.2898 (2)	0.8857 (3)	0.33946 (19)	0.0412 (8)	
C17	0.3125 (3)	0.7868 (3)	0.39402 (18)	0.0421 (8)	
C18	0.2400 (3)	0.6945 (3)	0.3864 (2)	0.0458 (8)	
C19	0.1198 (3)	0.6706 (3)	0.3228 (2)	0.0636 (10)	
H19A	0.1270	0.6179	0.2804	0.095*	
H19B	0.0852	0.7379	0.2938	0.095*	
H19C	0.0697	0.6405	0.3532	0.095*	
C20	0.4144 (3)	0.7692 (3)	0.4683 (2)	0.0487 (9)	
H20	0.4781	0.8178	0.4902	0.058*	
C21	0.4025 (3)	0.6688 (3)	0.5020 (2)	0.0503 (9)	
C22	0.4826 (4)	0.6117 (3)	0.5744 (3)	0.0648 (10)	
C23	0.2477 (3)	0.5162 (3)	0.4684 (2)	0.0763 (12)	
H23A	0.1777	0.5282	0.4845	0.115*	
H23B	0.3070	0.4786	0.5150	0.115*	
H23C	0.2274	0.4717	0.4165	0.115*	

### Atomic displacement parameters (Å^2^)

**Table:**

	*U*^11^	*U*^22^	*U*^33^	*U*^12^	*U*^13^	*U*^23^
S1	0.0588 (6)	0.0534 (6)	0.0508 (5)	0.0051 (5)	0.0180 (4)	−0.0076 (5)
F1	0.156 (2)	0.127 (2)	0.0683 (16)	−0.0037 (19)	0.0043 (16)	−0.0514 (16)
F2	0.0529 (15)	0.164 (3)	0.1049 (18)	−0.0014 (16)	−0.0008 (13)	0.0156 (18)
F3	0.0771 (15)	0.0648 (14)	0.0678 (13)	0.0198 (11)	−0.0099 (11)	−0.0011 (11)
F4	0.0955 (17)	0.0751 (16)	0.0794 (15)	−0.0045 (13)	0.0449 (13)	0.0115 (12)
F5	0.0856 (17)	0.0976 (19)	0.0857 (16)	0.0291 (14)	0.0254 (13)	−0.0200 (14)
F6	0.1210 (19)	0.0524 (14)	0.0784 (15)	−0.0257 (13)	0.0048 (13)	0.0062 (12)
F7	0.1296 (19)	0.0614 (14)	0.0532 (13)	−0.0118 (13)	0.0366 (13)	−0.0117 (11)
F8	0.0577 (13)	0.0680 (14)	0.0752 (14)	−0.0230 (11)	−0.0058 (10)	0.0032 (12)
N1	0.0634 (19)	0.0405 (17)	0.0494 (17)	−0.0112 (15)	0.0208 (15)	−0.0042 (15)
N2	0.102 (3)	0.081 (3)	0.075 (2)	0.010 (2)	0.002 (2)	0.016 (2)
C1	0.056 (2)	0.068 (3)	0.046 (2)	−0.007 (2)	0.0096 (18)	0.0015 (19)
C2	0.057 (3)	0.078 (3)	0.066 (3)	−0.015 (2)	0.003 (2)	0.018 (2)
C3	0.085 (3)	0.065 (3)	0.055 (3)	−0.022 (3)	−0.013 (2)	0.006 (2)
C4	0.104 (4)	0.064 (3)	0.052 (3)	−0.014 (3)	0.010 (3)	−0.015 (2)
C5	0.069 (2)	0.063 (3)	0.055 (2)	−0.009 (2)	0.017 (2)	−0.012 (2)
C6	0.055 (2)	0.049 (2)	0.038 (2)	−0.0065 (18)	0.0112 (17)	0.0029 (17)
C7	0.0455 (19)	0.047 (2)	0.0369 (18)	−0.0029 (16)	0.0097 (15)	−0.0031 (17)
C8	0.0425 (19)	0.052 (2)	0.0449 (19)	0.0078 (16)	0.0097 (16)	−0.0023 (18)
C9	0.0421 (19)	0.050 (2)	0.0345 (18)	0.0041 (17)	0.0083 (15)	−0.0031 (16)
C10	0.0448 (19)	0.053 (2)	0.0412 (19)	0.0045 (17)	0.0163 (15)	0.0009 (17)
C11	0.048 (2)	0.076 (3)	0.063 (2)	0.015 (2)	0.0135 (18)	0.000 (2)
C12	0.0367 (18)	0.050 (2)	0.0378 (19)	0.0023 (16)	0.0078 (15)	−0.0015 (17)
C13	0.044 (2)	0.057 (2)	0.048 (2)	0.0072 (18)	0.0083 (17)	0.0036 (19)
C14	0.055 (2)	0.046 (2)	0.060 (2)	−0.0015 (19)	0.0124 (19)	−0.0044 (19)
C15	0.053 (2)	0.050 (2)	0.045 (2)	−0.0128 (18)	0.0073 (17)	−0.0046 (19)
C16	0.0323 (17)	0.048 (2)	0.0430 (19)	−0.0066 (15)	0.0118 (15)	−0.0011 (17)
C17	0.0427 (19)	0.046 (2)	0.0364 (18)	−0.0034 (17)	0.0115 (15)	−0.0006 (16)
C18	0.0460 (19)	0.048 (2)	0.045 (2)	−0.0111 (18)	0.0171 (16)	−0.0070 (18)
C19	0.055 (2)	0.069 (3)	0.064 (2)	−0.023 (2)	0.0148 (18)	−0.013 (2)
C20	0.050 (2)	0.048 (2)	0.045 (2)	−0.0089 (17)	0.0101 (17)	−0.0056 (18)
C21	0.052 (2)	0.051 (2)	0.045 (2)	−0.0011 (19)	0.0112 (17)	−0.0002 (18)
C22	0.074 (3)	0.054 (2)	0.061 (3)	0.001 (2)	0.015 (2)	0.000 (2)
C23	0.098 (3)	0.056 (2)	0.079 (3)	−0.025 (2)	0.034 (2)	0.007 (2)

### Geometric parameters (Å, °)

**Table:**

S1—C10	1.713 (3)	C8—H8	0.9300
S1—C7	1.724 (3)	C9—C10	1.370 (4)
F1—C4	1.357 (4)	C9—C12	1.467 (4)
F2—C2	1.352 (4)	C10—C11	1.499 (4)
F3—C13	1.349 (3)	C11—H11A	0.9600
F4—C13	1.366 (4)	C11—H11B	0.9600
F5—C14	1.343 (4)	C11—H11C	0.9600
F6—C14	1.343 (4)	C12—C16	1.355 (4)
F7—C15	1.345 (4)	C12—C13	1.494 (4)
F8—C15	1.364 (3)	C13—C14	1.518 (4)
N1—C18	1.357 (4)	C14—C15	1.524 (5)
N1—C21	1.381 (4)	C15—C16	1.496 (4)
N1—C23	1.467 (4)	C16—C17	1.457 (4)
N2—C22	1.139 (4)	C17—C18	1.387 (4)
C1—C2	1.372 (5)	C17—C20	1.414 (4)
C1—C6	1.387 (4)	C18—C19	1.487 (4)
C1—H1	0.9300	C19—H19A	0.9600
C2—C3	1.359 (5)	C19—H19B	0.9600
C3—C4	1.367 (5)	C19—H19C	0.9600
C3—H3	0.9300	C20—C21	1.355 (4)
C4—C5	1.375 (5)	C20—H20	0.9300
C5—C6	1.383 (4)	C21—C22	1.422 (5)
C5—H5	0.9300	C23—H23A	0.9600
C6—C7	1.470 (4)	C23—H23B	0.9600
C7—C8	1.357 (4)	C23—H23C	0.9600
C8—C9	1.426 (4)		
			
C10—S1—C7	93.06 (15)	F4—C13—C12	111.3 (3)
C18—N1—C21	108.7 (3)	F3—C13—C14	112.0 (3)
C18—N1—C23	125.9 (3)	F4—C13—C14	109.0 (3)
C21—N1—C23	125.4 (3)	C12—C13—C14	105.3 (3)
C2—C1—C6	119.6 (4)	F5—C14—F6	107.0 (3)
C2—C1—H1	120.2	F5—C14—C13	109.4 (3)
C6—C1—H1	120.2	F6—C14—C13	114.9 (3)
F2—C2—C3	118.7 (4)	F5—C14—C15	109.1 (3)
F2—C2—C1	118.2 (4)	F6—C14—C15	113.0 (3)
C3—C2—C1	123.1 (4)	C13—C14—C15	103.3 (3)
C2—C3—C4	116.3 (4)	F7—C15—F8	105.5 (3)
C2—C3—H3	121.9	F7—C15—C16	114.3 (3)
C4—C3—H3	121.9	F8—C15—C16	111.1 (3)
F1—C4—C3	118.6 (4)	F7—C15—C14	112.1 (3)
F1—C4—C5	117.9 (4)	F8—C15—C14	108.7 (3)
C3—C4—C5	123.4 (4)	C16—C15—C14	105.0 (3)
C4—C5—C6	119.0 (4)	C12—C16—C17	130.5 (3)
C4—C5—H5	120.5	C12—C16—C15	109.8 (3)
C6—C5—H5	120.5	C17—C16—C15	119.6 (3)
C5—C6—C1	118.6 (3)	C18—C17—C20	106.7 (3)
C5—C6—C7	121.0 (3)	C18—C17—C16	128.1 (3)
C1—C6—C7	120.3 (3)	C20—C17—C16	125.1 (3)
C8—C7—C6	128.4 (3)	N1—C18—C17	108.3 (3)
C8—C7—S1	110.1 (2)	N1—C18—C19	121.6 (3)
C6—C7—S1	121.5 (2)	C17—C18—C19	130.0 (3)
C7—C8—C9	113.8 (3)	C18—C19—H19A	109.5
C7—C8—H8	123.1	C18—C19—H19B	109.5
C9—C8—H8	123.1	H19A—C19—H19B	109.5
C10—C9—C8	112.3 (3)	C18—C19—H19C	109.5
C10—C9—C12	124.3 (3)	H19A—C19—H19C	109.5
C8—C9—C12	123.3 (3)	H19B—C19—H19C	109.5
C9—C10—C11	129.4 (3)	C21—C20—C17	107.6 (3)
C9—C10—S1	110.7 (2)	C21—C20—H20	126.2
C11—C10—S1	119.8 (2)	C17—C20—H20	126.2
C10—C11—H11A	109.5	C20—C21—N1	108.6 (3)
C10—C11—H11B	109.5	C20—C21—C22	129.4 (3)
H11A—C11—H11B	109.5	N1—C21—C22	122.0 (3)
C10—C11—H11C	109.5	N2—C22—C21	178.8 (4)
H11A—C11—H11C	109.5	N1—C23—H23A	109.5
H11B—C11—H11C	109.5	N1—C23—H23B	109.5
C16—C12—C9	129.1 (3)	H23A—C23—H23B	109.5
C16—C12—C13	110.4 (3)	N1—C23—H23C	109.5
C9—C12—C13	120.3 (3)	H23A—C23—H23C	109.5
F3—C13—F4	105.0 (3)	H23B—C23—H23C	109.5
F3—C13—C12	114.3 (3)		
			
C6—C1—C2—F2	179.6 (3)	C12—C13—C14—F6	146.1 (3)
C6—C1—C2—C3	−0.6 (6)	F3—C13—C14—C15	147.3 (3)
F2—C2—C3—C4	−179.6 (3)	F4—C13—C14—C15	−97.0 (3)
C1—C2—C3—C4	0.6 (6)	C12—C13—C14—C15	22.5 (3)
C2—C3—C4—F1	−177.8 (3)	F5—C14—C15—F7	−32.5 (4)
C2—C3—C4—C5	0.2 (6)	F6—C14—C15—F7	86.4 (4)
F1—C4—C5—C6	177.1 (3)	C13—C14—C15—F7	−148.8 (3)
C3—C4—C5—C6	−0.9 (6)	F5—C14—C15—F8	−148.8 (3)
C4—C5—C6—C1	0.9 (5)	F6—C14—C15—F8	−29.9 (4)
C4—C5—C6—C7	−176.3 (3)	C13—C14—C15—F8	94.9 (3)
C2—C1—C6—C5	−0.2 (5)	F5—C14—C15—C16	92.2 (3)
C2—C1—C6—C7	177.0 (3)	F6—C14—C15—C16	−148.9 (3)
C5—C6—C7—C8	150.7 (3)	C13—C14—C15—C16	−24.1 (3)
C1—C6—C7—C8	−26.4 (5)	C9—C12—C16—C17	−6.6 (5)
C5—C6—C7—S1	−28.1 (4)	C13—C12—C16—C17	178.4 (3)
C1—C6—C7—S1	154.7 (3)	C9—C12—C16—C15	172.1 (3)
C10—S1—C7—C8	0.5 (3)	C13—C12—C16—C15	−2.9 (4)
C10—S1—C7—C6	179.5 (3)	F7—C15—C16—C12	140.7 (3)
C6—C7—C8—C9	−179.4 (3)	F8—C15—C16—C12	−99.9 (3)
S1—C7—C8—C9	−0.5 (3)	C14—C15—C16—C12	17.5 (4)
C7—C8—C9—C10	0.3 (4)	F7—C15—C16—C17	−40.4 (4)
C7—C8—C9—C12	179.3 (3)	F8—C15—C16—C17	78.9 (4)
C8—C9—C10—C11	179.3 (3)	C14—C15—C16—C17	−163.7 (3)
C12—C9—C10—C11	0.3 (5)	C12—C16—C17—C18	−40.4 (5)
C8—C9—C10—S1	0.1 (3)	C15—C16—C17—C18	141.1 (3)
C12—C9—C10—S1	−178.9 (2)	C12—C16—C17—C20	141.8 (3)
C7—S1—C10—C9	−0.3 (3)	C15—C16—C17—C20	−36.7 (4)
C7—S1—C10—C11	−179.6 (3)	C21—N1—C18—C17	0.0 (4)
C10—C9—C12—C16	−52.0 (5)	C23—N1—C18—C17	179.1 (3)
C8—C9—C12—C16	129.1 (3)	C21—N1—C18—C19	−177.9 (3)
C10—C9—C12—C13	122.7 (3)	C23—N1—C18—C19	1.2 (5)
C8—C9—C12—C13	−56.2 (4)	C20—C17—C18—N1	−0.7 (3)
C16—C12—C13—F3	−136.2 (3)	C16—C17—C18—N1	−178.8 (3)
C9—C12—C13—F3	48.3 (4)	C20—C17—C18—C19	177.0 (3)
C16—C12—C13—F4	105.1 (3)	C16—C17—C18—C19	−1.1 (6)
C9—C12—C13—F4	−70.5 (4)	C18—C17—C20—C21	1.1 (4)
C16—C12—C13—C14	−12.9 (4)	C16—C17—C20—C21	179.3 (3)
C9—C12—C13—C14	171.6 (3)	C17—C20—C21—N1	−1.1 (4)
F3—C13—C14—F5	31.2 (4)	C17—C20—C21—C22	177.4 (3)
F4—C13—C14—F5	146.9 (3)	C18—N1—C21—C20	0.7 (4)
C12—C13—C14—F5	−93.6 (3)	C23—N1—C21—C20	−178.4 (3)
F3—C13—C14—F6	−89.1 (4)	C18—N1—C21—C22	−178.0 (3)
F4—C13—C14—F6	26.6 (4)	C23—N1—C21—C22	3.0 (5)
